# Assessing the Challenges of Managing Pharmacy Services in Public Hospitals in Erbil City

**DOI:** 10.5812/ijpr-162251

**Published:** 2025-08-03

**Authors:** Sanar Ilias Kamal, Kemal M Surji

**Affiliations:** 1 Department of Pharmacology and Toxicology, Hawler Medical University, Erbil, Iraq

**Keywords:** Pharmacy Services, Challenges, Public Hospitals, and Quality of Care

## Abstract

**Background:**

Pharmacy services in public hospitals are crucial for improving patients’ health and effectively managing diseases. Previously, these services were limited to dispensing medications and counseling; they have now expanded to include a more integrated, patient-centered approach that actively supports clinical decisions and enhances treatment outcomes.

**Objectives:**

This study aims to identify and explore the pharmacy services provided in Erbil’s public hospitals, highlighting the primary challenges encountered and the strategies employed to address them.

**Methods:**

An exploratory sequential mixed-methods approach was employed, combining qualitative and quantitative data collection methods. The qualitative phase consisted of semi-structured, in-depth, face-to-face interviews with nine pharmacy heads in Erbil’s public hospitals, which were analyzed using Braun and Clarke’s six-step method to identify themes. This phase was followed by a quantitative phase, which involved a structured, closed-ended questionnaire distributed to 250 pharmacy staff, with the data analyzed using SPSS 24.

**Results:**

In the qualitative phase, five main themes emerged, identifying the state of pharmacy services and the significant challenges that directly impact the quality of care, including frequent drug shortages (N = 9), staffing shortages (N = 5), technological limitations (N = 8), and regulatory challenges (N = 9). In the quantitative phase, data from 212 completed questionnaires revealed the range of pharmacy services provided by public hospitals, including medication dispensing (88.2%), counseling (42.85%), inventory management (72.64%), and clinical services (48.11%), as well as the challenges faced in delivering these services effectively. Therefore, systematic strategies are needed to overcome these barriers.

**Conclusions:**

The findings of this study indicate that the provision of pharmacy services within Erbil’s public hospitals faces numerous challenges, indicating the need for increased investments in public hospitals, improved regulatory support, and the adoption of technological resources, all of which are essential for the advancement of pharmacy services and the improvement of patient outcomes.

## 1. Background

Pharmacy services in public hospitals play a vital role in enhancing patients’ health and managing diseases effectively by ensuring access to necessary treatments and improving overall healthcare quality (1, 2). Previously, these services were primarily focused on dispensing prescriptions and counseling patients (3). However, in recent years, the roles of pharmacists have expanded to include more patient-centered care and clinical services. This evolution has enabled pharmacists to actively improve health outcomes, demonstrating their importance in enhancing healthcare (1, 4-6). Considering that nearly every hospital patient requires medication, effective pharmacy management and addressing management challenges are essential to ensuring high-quality healthcare delivery (7, 8).

From a global and regional perspective, particularly in resource-limited countries, data indicate that numerous challenges affect the quality of healthcare provided, including medication shortages, which can lead to delays or compromised patient care (9-12). Pharmacist shortages and inadequate training for pharmacists further limit the quality of clinical services provided in hospitals (13-17). Moreover, poor integration of health information technology significantly reduces the efficiency and safety of pharmacy operations (18-21). Finally, budgetary constraints are considered the basic root of numerous challenges, limiting access to resources and adversely impacting healthcare quality (22-25). Consequently, these challenges in healthcare services significantly impact the delivery of clinical services, patient outcomes, and the overall quality of care in hospitals.

This study addresses a significant gap in healthcare systems research in Iraq by focusing particularly on pharmacy service delivery in public hospitals in Erbil. Previous studies in the region have primarily concentrated on general health system challenges, with limited investigation of pharmacy services. This study offers a comprehensive understanding of pharmacy services and their challenges, employing a mixed-methods approach that combines frontline staff experiences with organizational-level analysis. Additionally, it provides extensive knowledge of regional pharmacy practices and presents a unique framework for systematic research in low-resource settings. These contributions distinguish the study from previous research and underscore its relevance for local health policy reform and broader international comparisons.

## 2. Objectives

This study aims to explore the current state of pharmacy services at major public hospitals in Erbil city, identify key challenges impacting their management, and propose strategies to improve these services and enhance patient-centered care. The findings will support policymakers in enhancing hospital services and healthcare outcomes.

## 3. Methods

### 3.1. Study Design

This study used a descriptive, cross-sectional approach employing an exploratory sequential mixed-methods design, which began with qualitative data collection and was followed by a quantitative phase. The qualitative phase consisted of one-on-one, semi-structured interviews with pharmacy heads across major public hospitals in Erbil City. These interviews were organized into five main sections aimed at exploring service delivery practices, significant challenges, and perceived barriers to effective pharmacy management. The qualitative findings directly guided the development of the quantitative questionnaire. Key themes were converted into 30 structured, closed-ended questions to ensure contextual significance, content validity, and reflection of staff experiences. The resulting survey was distributed to pharmacy staff to assess the prevalence and challenges identified in the qualitative phase.

Before conducting data collection, several measures were taken to minimize interviewer bias across both study phases. The interviewer maintained a neutral, non-judgmental environment, while the questionnaire was designed to be self-administered, allowing participants to complete it privately without influence from the data collector. To reduce social desirability bias, participants were assured of full anonymity and confidentiality, with no identifying information being recorded.

### 3.2. Study Setting and Data Collection

The study was conducted between October 2024 and February 2025, including both qualitative and quantitative phases across nine major hospitals in Erbil City.

#### 3.2.1. The Qualitative Phase

An open-ended, semi-structured interview guide was developed and reviewed by four academic pharmacists for content validity. It was subsequently pilot tested with two pharmacists to enhance interviewing skills, promote a nonjudgmental environment, and ensure adherence to the protocol. After obtaining consent, interviews were conducted in both Kurdish and Arabic to ensure participants’ comfort and clarity of expression. Each interview lasted between 60 and 80 minutes. The interview transcripts were then translated into English to facilitate collaborative analysis with the co-researcher, meet publication language requirements, and ensure consistent terminology during coding and thematic analysis. Bilingual researchers evaluated the translation to ensure cultural relevance and appropriateness. A forward–backward translation method was utilized, adhering to the guidelines outlined by Brislin (1970), to ensure conceptual and linguistic similarity. The accuracy of the translation was further cross-validated using Google Translate as a supplementary tool (26).

#### 3.2.2. The Quantitative Phase

The quantitative phase involved the development of a structured, closed-ended questionnaire designed to replicate the questions of the qualitative interviews. The questionnaire was translated into Kurdish and Arabic to ensure comprehension for pharmacy staff with different language preferences. The translated version was evaluated by bilingual experts for cultural and linguistic accuracy. The initial draft was reviewed by four academic pharmacists, including clinical pharmacists, a hospital administrator, and two academic professionals in health management. They assessed the face validity of each item by examining its clarity, wording, and appropriateness. Subsequently, content validity was conducted using a structured rating form; they evaluated each item for relevance on a four-point scale (1 = not relevant, 4 = highly relevant). The Content Validity Index (CVI) was calculated for each item (I-CVI) and the entire scale (S-CVI). Items with I-CVI values below 0.78 were revised or removed based on expert feedback. The resulting S-CVI/Ave value of the revised instrument exceeded the recommended threshold of 0.90, indicating high content validity.

Finally, the refined questionnaire was pilot-tested with 20 pharmacy employees (who were excluded from the final data collection) to assess the estimated time for completion, relevance, and clarity. Minor modifications to the layout and phrasing were implemented in response to feedback from the pilot participants.

### 3.3. Participants and Sampling Strategies

For the qualitative phase, a purposive sampling approach was employed. The study targeted all nine public hospitals in Erbil with centralized pharmacies. From each hospital, the head of the pharmacy department was selected based on their managerial positions and direct supervision of pharmacy operations. Given the limited number of public hospitals in Erbil with centralized pharmacies, this sample represents a comprehensive perspective on service provision, key challenges, and regulatory issues.

In the quantitative phase, convenience sampling was used. The estimated total number of pharmacy staff across the nine targeted public hospitals was approximately 300. A total of 250 questionnaires were self-distributed to pharmacy staff during their working hours. Of these, 212 valid and fully completed responses were obtained and included in the final analysis. The participants brought diverse experiences and challenges in areas like internal and external pharmacy, hospital inventory, clinical services, and procurement committees.

### 3.4. Data Analysis

Thematic analysis was employed to analyze the interviews, following Braun and Clarke’s six-step method, which includes familiarization, generating codes, theme development, refinement, defining and labeling themes, and writing the results (27). For the quantitative results, data were coded in Excel and analyzed in SPSS (version 24) using descriptive statistics (frequencies and percentages). Additionally, one-way ANOVA was conducted to compare the years of experience and the perception of pharmacy care quality.

### 3.5. Ethical Approval

The study was approved by the Research Ethics Committee of Hawler Medical University, College of Pharmacy (letter No.: 604, October 20, 2024). The study’s aims, objectives, and confidentiality assurance were clearly explained prior to data collection. To ensure transparency and privacy during the qualitative phase, verbal consent was obtained and documented in a consent tracking log. This log included the participant’s anonymized code, the date of consent, and verification that the information had been reviewed with the participant. No audio recordings of consent were made; however, all consent information was recorded promptly after verbal agreement.

In the quantitative phase, participants read the information and agreed to voluntarily consent to participate. Additionally, all interviews and questionnaires were conducted anonymously to ensure participants’ confidentiality.

## 4. Results

These findings examine pharmacy services in Erbil’s public hospitals, highlighting primary challenges based on qualitative and quantitative data collected through interviews and questionnaires. Table 1 illustrates the alignment between qualitative and quantitative findings, enhancing the integration of results across methods.

**Table 1.** Integration of Interview and Survey Findings on Key Aspects of Pharmacy Service Delivery

Themes	Qualitative Result	Quantitative Result	Interpretation
**Provided pharmacy services**	Heads of pharmacy reported providing medication dispensing (N = 9), patient counseling (N = 8), inventory management (N = 8), and clinical services (N = 5).	Pharmacy staff reported that they provide medication dispensing (88.2%), patient counseling (42.85%), inventory management (72.64%), and clinical services (48.11%).	Both findings confirmed that medication dispensing is the primary service. However, clinical and counseling services are inconsistently provided across hospitals.
**Recent developments in provided services**	Some pharmacy heads identified positive impacts from recent service development (N = 5), while others noted a lack of observable improvements (N = 4).	The majority of staff reported that services are rarely reviewed (63.2%), with only a few indicating monthly (3.3%) or annual (22.2%) service updates.	Findings indicate minimal and uneven development of pharmacy services. Quantitative results confirm qualitative concerns about limited service improvement.
**Patient satisfaction**	Pharmacy heads identified a significant lack in patient feedback systems (N = 9). While patients are considered as satisfied with essential medication availability (N = 6), dissatisfaction was noted regarding other pharmacy services (N = 3).	The majority of pharmacy staff stated that patient feedback is rarely/never collected (71.7%). Most patients state they feel neutral (53.8%) or satisfied (30.2%) with services.	The lack of feedback mechanisms hinders accurate assessment of patient satisfaction. Perceived satisfaction remains low to moderate.
**Key challenges **	Pharmacy heads identified several persistent challenges, including staff shortages (N = 9), medication shortages (N = 5), budgetary constraints (N = 7), technological limitations (N = 8), lack of CPD programs (N = 7), and delayed procurement/quality assurance regulations (N = 7) as the main challenges.	Pharmacy staff reflected these findings: Staff shortage (74.05%), medication shortage (66.5%), budgetary constraints (84.4%), technological limitations (78.7%), lack of CPD programs (39.2%), and delayed procurement/quality assurance regulations (88.7%).	All challenges identified by interviewers are highly supported by survey data. The quantitative challenge of the lack of CPD programs is less prioritized than technological limitations and budgetary constraints that are strongly reported by both findings.

Abbreviation: CPD, continuous professional development.

### 4.1. Qualitative Analysis

The analysis of interviews with nine pharmacy heads resulted in five themes, each with subsequent subthemes. (To ensure anonymity, the nine heads of pharmacy interviewed have been referred to as P1 through P9 throughout this analysis.) Appendix 1 in Supplementary File provides a detailed overview of the thematic analysis, including themes, sub-themes, codes, and quotations.

#### 4.1.1. Theme 1: Provided Pharmacy Services

All participants (N = 9) indicated that medication dispensing is a primary service, serving both inpatients and outpatients. "Our hospital has many pharmacies, such as internal, emergency, consultation, and artificial kidney pharmacies, and they are responsible for supplying patients with necessary medications." (P7). Most participants (N = 8) stated that they provided guidance on medication use in addition to dispensing. Instructions included frequency of intake, timing related to meals, and treatment duration. "We provide patients with prescribed medications and instruct them on their proper usage throughout our external or consultation pharmacy." (P2).

Inventory management is universally practiced (N = 8) by pharmacy staff, encompassing procurement, receipt, storage, and distribution of pharmaceutical and other medical supplies to pharmacies and hospital departments. "Our staff manage the procurement, receipt, and distribution of all pharmaceuticals and medical supplies throughout the hospital." (P4). Finally, only a few hospitals (N = 5) provide clinical services. However, the offered clinical services consisted of the most basic clinical activities, suggesting underutilization of pharmacists’ clinical roles. "Clinical pharmacists are observing the process of ordering the prescribed drugs from internal pharmacies and supervising the drug administration process." (P1).

#### 4.1.2. Theme 2: Recent Developments in Provided Services

More than half (N = 5) of the participants reported improvements in pharmacy services, which included establishing new storage (N = 1), expanding the range of available medications (N = 3), offering full-time internal pharmacy services (N = 1), and renovating existing pharmacies alongside the development of new ones (N = 2). "The provided services have enhanced in recent years, mainly owing to the accessibility of several treatments that were previously unaffordable." (P2).

#### 4.1.3. Theme 3: Patient Satisfaction

All interviewed pharmacy heads (N = 9) indicated that there are no standard protocols for collecting patients’ feedback about pharmacy services. "Unfortunately, our hospital doesn’t have any formal mechanism to collect patients' feedback officially." (P1). Despite the lack of formal documentation, some participants (N = 6) considered that patients are generally satisfied with the availability of essential medications. Still, they are less confident in other direct patient-centered care services. "Our patients receive most of the essential drugs; however, when it comes to care, counseling, and other clinical services, we are not the best." (P7).

#### 4.1.4. Theme 4: Key Challenges, Underlying Causes, and Adaptation Strategies

##### 4.1.4.1. Medication Shortage

All pharmacies (N = 9) reported shortages in medical supplies and medications due to factors like supply chain deficiencies (N = 7), delays in quality control approval tests (N = 7), unequal distribution of drugs among hospitals (N = 2), and patient overload (N = 2). Strategies used by heads of pharmacies to adapt to these shortages included seeking drugs from other hospitals (N = 8), creating a list of the available medicines and providing alternative options to prescribers (N = 2), or asking the patient to get the drug from external sources (N = 8). "Medications and medical supplies are in short supply, particularly antibiotics, which are limited to basic older generations." (P4). "In case of an urgent shortage, we maintain strong communication with other hospitals to cover gaps." (P2). "Providing physicians with the currently available drug list would sometimes help to overcome drug shortages." (P8). "In case of unsolved drug shortages, we request patients' relatives to obtain it from external sources." (P8).

##### 4.1.4.2. Staff Shortages

Several pharmacy heads noted that both staff shortages (N = 5) and low engagement among existing staff (N = 4) compromise the quality of care. These issues stem from various factors, including the uneven distribution of pharmacists by patient volume and workload, causing them to leave overloaded facilities for those with less workload (N = 8). Additionally, most employees were female and struggled to balance work and family, limiting their availability for work (N = 3). The economic instability and delays in monthly wages in the region further exacerbate the shortages (N = 8). To overcome the workload resulting from staff shortages, which aligns with the financial state and delays in monthly salaries, pharmacy heads have reduced employees’ working days through the adoption of flexible schedules; however, the effectiveness of this strategy remains questionable. "Staff shortages significantly reduce the range of pharmacy services. If we had more employees, we would be able to deliver a wider range of services more effectively." (P5). "Most of our pharmacists are female, and they are more committed to their families; this is one of the reasons behind staff shortages." (P2). "Due to the economic situation and wage shortages, we have had to reduce staff working days and hours to balance the workload resulting from staff shortages." (P3).

##### 4.1.4.3. Technological Limitations

The majority of pharmacy heads (N = 8) reported that they operate without technological support, which increases employee workload and heightens the likelihood of errors. As a strategy to adapt to these shortages, pharmacy staff are conducting all operations manually, which has led to pharmacists being more occupied with logistics tasks rather than focusing on clinical services. Financial constraints were identified as the primary barrier to adopting technology (N = 8). "Technology is extremely required, as we are suffering from a lack of technological facilities." (P5). "Technology shortage is covered by handling the process manually." (P7). "The primary barrier to technology implementation is financial constraints, as these technologies require substantial funding." (P2).

##### 4.1.4.4. Regulatory Challenges

All nine pharmacy heads have confirmed that the regulations governing pharmacy management in public hospitals are inadequate and negatively impact the quality of pharmacy services. "In my opinion, the most significant shortage regarding pharmacists is the absence of enough regulations." (P4). "There are no specific regulations that deserve to be mentioned affecting the quality of services provided." (P2). The majority of pharmacy heads (N = 7) reported encountering budgetary constraints, identifying them as the primary cause of widespread shortages. Additionally, they highlighted a significant lack of continuous professional development (CPD) programs for staff, limiting pharmacists’ opportunities for skill enhancement (N = 7). Furthermore, procurement and quality assurance regulations were stated as contributing to medication supply delays and reduced availability in pharmacies (N = 7). "Budget constraints affect most of the other challenges, such as drug shortages and technological limitations." (P8). "Pharmacists face a severe shortage in scientific training and other professional development programs, and the main reason behind that is budgetary constraints." (P7). "Medication shortages are due to various factors, including the regulatory framework of the procurement process and the prolonged process of quality control approval tests." (P2).

#### 4.1.5. Theme 5: Future Recommendations for Overcoming Pharmacy-Related Challenges

This theme gathered pharmacy managers’ suggestions for improving services through unified regulations, such as standard job descriptions that identify pharmacists’ roles and responsibilities (N = 8), including their annual enrollment in CPD programs (N = 5). "The primary regulation that is crucial to be developed and implemented is the job description for pharmacists, which will unify their efforts in all public hospitals and reduce the disparities between the roles of pharmacists within the hospitals." (P8). "Addition of regulations about training and developmental programs that should be mandatory for pharmacists and pharmacy staff to renew their licenses annually and get updated about the advances in health science." (P8).

Additionally, regulations for technology implementation enhance service quality (N = 8) and impose strict inventory standards to ensure the proper and safe storage of supplies following standard regulations (N = 3). "Insufficient facilities for the management of drugs and medical supplies, coupled with inadequate space and the essential standards for effective storage." (P3). "Regulation of mandatory implementing software programs is highly required to enhance the quality of service." (P2).

Finally, strict regulations regarding the management of controlled substances (N = 3) are needed, as such laws would prevent or reduce the rate of abuse of controlled substances. "The second regulation that needs to be implemented is strict regulations about the management of controlled substances in public hospitals to control the increased rates of abuse." (P8).

### 4.2. Quantitative Analysis

A total of 212 out of 250 distributed questionnaires were completed, yielding a response rate of 84.8%. The sample consisted predominantly of females (61.3%), with ages mostly between 26 - 35 and 36 - 45 years (40.1% each). Most participants held bachelor's degrees in pharmacy (83%), while a significant portion had over ten years of experience (59%), as shown in Table 2. 

**Table 2.** Sociodemographic Characteristics of the Participants ^a^

Variables	Values
Gender	
Male	82 (38.7)
Female	130 (61.3)
**Age (y)**	
18 - 25	13 (6.1)
26 - 35	85 (40.1)
36 - 45	85 (40.1)
46 - 55	28 (13.2)
> 56	1 (0.5)
**Educational background**	
Diploma	30 (14.2)
BSc.	176 (83)
Msc.	4 (1.9)
PhD.	2 (0.9)
**Years of experience**	
1 - 3	5 (2.4)
4 - 6	28 (13.2)
7 - 10	54 (25.5)
More than 10	125 (59)

Abbreviations: BSc., Bachelor of Science; MSc., Master of Science; PhD., Doctor of Philosophy.

^a^ Values ae expressed as No. (%).

#### 4.2.1. Provided Pharmacy Services

As shown in Table 3, the majority of participants were involved in medication dispensing (88.2%) and inventory tracking (72.6%), while 48.11% provided clinical services and 42.85% offered patient counseling. Regarding service quality and patient satisfaction, 53.8% of participants rated services as good, yet 24.5% considered them fair. The majority indicated that patient feedback is rarely or never collected (71.7%), hindering the ability to assess the level of patient satisfaction. Additionally, 80.2% of respondents reported that they lack the necessary technological resources to provide pharmacy services, indicating a need for improvement. Approximately 43.9% of pharmacies served over 100 patients per day, with the majority reporting waiting times of 5 - 10 minutes.

**Table 3.** Pharmacy Staff Perception Toward the Current State of Pharmacy Services Offered Within Public Hospitals ^a^

Variables	Values
What types of pharmacy services are currently offered in your facility?	
Medication dispensing	187 (88.2)
Clinical pharmacy	102 (48.11)
Patient counseling	93 (42.85)
Drug utilization review	23 (10.89)
Inventory tracking	154 (72.64)
**How would you rate the overall quality of pharmacy services in your hospital?**	
Poor	7 (3.3)
Fair	52 (24.5)
Good	114 (53.8)
Very good	32 (15.1)
Excellent	7 (3.3)
**How frequently are pharmacy services reviewed and updated in your hospital?**	
Monthly	7 (3.3)
Quarterly	13 (6.1)
Semi-annual	11 (5.2)
Annually	47 (22.2)
Rarely	134 (63.2)
**What technologies are currently utilized in your pharmacy services?**	
Electronic health records (EHR)	2 (0.9)
Pharmacy management software	39 (18.4)
Tele pharmacy	1 (0.5)
None of above	170 (80.2)
**How many patients, on average, does your pharmacy serve daily?**	
Less than 50	37 (17.5)
50 - 100	82 (38.7)
More than 100	93 (43.9)
**What is the average wait time for a patient to receive their medication? (min)**	
Less than 5	75 (35.4)
5 - 10	109 (51.4)
11 - 20	20 (9.4)
More than 20	8 (3.8)
**How often do you receive feedback from patients regarding pharmacy services?**	
Daily	6 (2.8)
Weekly	4 (1.9)
Monthly	4 (1.9)
Occasionally	46 (21.7)
Rarely/never	152 (71.7)

^a^ Values ae expressed as No. (%).

To examine whether years of work experience impact the perception of pharmacy staff toward the quality of pharmacy care delivery, the results revealed no statistically significant difference in the perceived quality of care across the different experience groups, F(4,206) = 0.753, P = 0.557. Since the P-value exceeds the conventional threshold of 0.05, the null hypothesis is to be rejected. This implies that the quality of services provided in public hospitals is not significantly influenced by the number of years of experience that pharmacy employees have.

#### 4.2.2. Key Challenges Experienced

Table 4 presents key obstacles faced by pharmacy staff, including technology shortages (78.7%), staff shortages (74.05%), and medication shortages (66.5%). Procurement and quality assurance (88.7%), along with budgetary constraints (84.4%), were identified as the main regulatory challenges.

**Table 4.** Key Challenges Impacting the Management of Pharmacy Services ^a^

Challenges	Values
**Staff shortage**	157 (74.05)
**Medication shortage**	141 (66.5)
**Technology shortage**	167 (78.7)
**Regulatory challenges**	
Budgetary constraints	179 (84.4)
Procurement and quality assurance	188 (88.7)
Inventory management	90 (42.5)
Controlled substance management	146 (68.9)
Staffing and training requirements	83 (39.2)

^a^ Values ae expressed as No. (%).

## 5. Discussion

This section discusses the key findings related to pharmacy services and highlights the primary challenges in managing services in Erbil’s public hospitals.

### 5.1. Current State of Pharmacy Services and Recent Improvements

Pharmacy services at public hospitals in Erbil city primarily focus on dispensing and basic counseling, with limited clinical services, indicating an underutilization of pharmacists’ clinical skills (Table 3). The primary factors contributing to the shortages of clinical pharmacy services are staff shortages, insufficient training, and heavy workload. Similar results were observed in a study conducted across Nigerian hospitals, where several factors contributing to the limited provision of clinical services were identified, including staff shortages, inadequate clinical education and training, and lack of specialization in practices (28).

A continuous quality improvement approach is crucial for enhancing the quality of pharmacy services; however, its implementation remains limited in most hospitals due to budget constraints, resistance to change, poor accountability, low staffing engagement, and weak regulatory frameworks (23). Research has shown that medication availability, waiting times, service quality, and pharmacist-patient interaction influence patient satisfaction (29-31). The recorded patient waiting time to receive pharmacy services in Erbil’s public hospitals ranged from 5 to 10 minutes, which is below the average 10 to 30 minute range reported in previous studies associated with reasonable patient satisfaction (4, 32, 33). The absence of structured feedback collection programs, alongside waiting times and drug shortages, underscores the need for a systematic approach to evaluating actual patient satisfaction.

However, it is important to emphasize that the primary objective of this study was not to assess the overall quality of care. Despite the inclusion of a single-item question to capture general perceptions, these findings should be interpreted with caution and are not intended for use in organizational planning or policymaking.

### 5.2. The Challenges of Delivering High-Quality Pharmaceutical Services

Numerous challenges heavily impacted the provision of high-quality pharmacy services. Findings from the current study (Table 4) indicate that 78.7% of pharmacy staff, as well as all pharmacy heads (N = 9), identified a shortage of technology as a major challenge. This finding raises serious concerns among pharmacy care providers and emphasizes the urgent need to improve technological infrastructure to support effective pharmacy care delivery. Evidence from the literature suggests that this shortage significantly compromises the efficiency and overall quality of pharmacy services (21, 34-36).

In addition to technological limitations, pharmacy staff identified several other challenges affecting service delivery. Notably, medication shortages were reported by 66.5% of respondents, while 74% highlighted staff shortages as a critical challenge. Furthermore, budgetary constraints, along with procurement and quality assurance systems, were emphasized as major regulatory challenges.

Each of the identified challenges exacerbates the others and negatively impacts the quality of patient care. For example, the implementation of technological resources is highly affected by challenges such as budgetary constraints, regulatory gaps, and continued reliance on traditional manual tasks (37, 38). On the other hand, the lack of technology further intensifies staffing shortages, as time-consuming manual tasks reduce pharmacists’ ability to focus on clinical responsibilities and patient safety, ultimately compromising the quality of care (39). Furthermore, challenges in procurement policies have been shown to contribute to medication shortages (40, 41).

Additionally, the lack of structured programs to enhance the clinical skills and professional performance of pharmacy staff contributes to the underutilization of their clinical roles. Several barriers affect the adoption of these programs, including insufficient resources, lack of motivation, regulatory gaps, and time constraints (15-17, 42). Low-resource developing countries, such as Iraq, Lebanon, Egypt, Sudan, Iran, Afghanistan, and Ethiopia, face similar challenges. These include medication and staff shortages, insufficient training for pharmacists, budgetary constraints, and regulatory gaps, all of which significantly impact the quality and effectiveness of pharmacy services.

For instance, research conducted in Egypt and Sudan has indicated that pharmacists’ ability to provide high-quality clinical services is limited by the lack of structured training and educational programs, pharmacist shortages, and the absence of clear job descriptions (10, 11, 14, 16, 22, 43, 44). These findings are consistent with the current study’s results and emphasize the significance of investing in workforce development to enhance clinical care in public hospitals.

Therefore, to overcome or minimize the effect of these challenges, actionable strategies are necessary, including standardizing job descriptions, implementing clinical training programs, investing in technological infrastructure to replace manual processes, rearranging procurement processes, and assigning specific budgets by stakeholders, all aimed at enhancing the quality, efficiency, and consistency of pharmacy care services.

### 5.3. Conclusions

This study examined the current state of pharmacy services in Erbil’s public hospitals, identifying key challenges that affect the quality, efficiency, and scope of service. Through a mixed-methods approach, it highlights primary challenges such as technological, medication, and staff shortages, in addition to regulatory challenges. While most hospitals are trying to address these shortages, other services, such as clinical services and CPD programs, remain underdeveloped.

The findings emphasize an urgent need for the Ministry of Health to prioritize standardized protocols, employee development, technological integration, and supportive regulatory frameworks. Policymakers and hospital administrators can easily implement these practical recommendations to enhance the quality of care in low-resource settings. Additionally, this study addresses a major gap in region-specific healthcare research by conducting one of the first empirical investigations on pharmacy operations in the region.

### 5.4. Implications of the Study

This study reveals critical gaps in pharmacy services in Erbil’s public hospitals, driven by systematic challenges such as staffing shortages, medication shortages, inadequate technology, and regulatory gaps. By integrating qualitative and quantitative data, this study offers a clear understanding of how these challenges impact the quality of care. Practically, the findings emphasize the urgent need for policy reforms to standardize pharmacy roles, invest in technological infrastructure, and expand training opportunities. Scholarly, the study contributes to the limited regional research and provides a framework for assessing pharmacy performance in low-resource settings. These insights not only guide local strategies but also provide applicable lessons for similarly limited healthcare systems globally.

### 5.5. Limitations

This study has several limitations that should be acknowledged. First, the quantitative data were obtained through self-reported questionnaires, which, despite assurances of anonymity, may have influenced responses due to recall or social desirability bias. Additionally, the gender imbalance in the quantitative sample (with the majority of participants being female) may have introduced gender-related bias, particularly in findings related to work-life balance and scheduling.

Second, although a standardized interview guide was used to reduce variability in the qualitative phase, interviewer influences may still have affected participants’ responses. Despite the utilization of a neutral, non-judgmental setting, respondents may have been hesitant to share critical views due to perceived professional risks. Moreover, this study was limited to public hospitals in Erbil City, which may limit the generalizability of the findings. As a result, the outcomes may not fully reflect pharmacy services in private sector hospitals or more rural healthcare facilities.

Finally, the exclusive focus on pharmacy staff, without including perspectives from other stakeholders such as hospital managers and patients, may have limited the depth and diversity of insight into the overall state of pharmacy care. In addition, while the study included a single-item question on perceived quality of care, this was not a primary objective. As such, related findings should not be used for policymaking or planning purposes, and future research should consider validated multi-item tools for a more reliable assessment of quality of care.

ijpr-24-1-162251-s001.pdf

## Data Availability

The dataset presented in the study is available on request from the corresponding author during submission or after publication. The data are not publicly available due to ethical considerations and the maintenance of participants' anonymity.
