# Supplementary material for: Assessing the Challenges of Managing Pharmacy Services in Public Hospitals in Erbil City
Source: Iran J Pharm Res. 2025 Aug 3;24(1):e162251. doi: 10.5812/ijpr-162251 (PMC12523809; doi:10.5812/ijpr-162251)
Supplement: ijpr-24-1-162251-s001.pdf [file ijpr-24-1-162251-s001.pdf]

Appendix 1.The estimated total number of pharmacy staff in the targeted hospitals

**List of Hospitals**	**Estimated number of pharmacy staff**	**Distributed questionnaire**	**Completed / Accepted Questionnaires**
Hospital **A**	24	20	20
Hospital **B**	27	25	20
Hospital **C**	35	34	30
Hospital **D**	35	28	24
Hospital **E**	6	4	4
Hospital **F**	98	76	62
Hospital **G**	10	10	10
Hospital **H**	50	38	31
Hospital **I**	15	15	11
**Total Number**	**300***	**250**	**212**

*The estimated number also accounts for pharmacy staff currently on leave due to maternity or other personal circumstances.
